# *N*-acetylcysteine for non-paracetamol drug-induced liver injury: a systematic review protocol

**DOI:** 10.1186/s13643-015-0075-6

**Published:** 2015-06-12

**Authors:** Mohamed Farouk Chughlay, Nicole Kramer, Mahmoud Werfalli, Wendy Spearman, Mark Emmanuel Engel, Karen Cohen

**Affiliations:** Division of Clinical Pharmacology, Department of Medicine, Faculty of Health Sciences, University of Cape Town, Anzio Road, Observatory 7925 Cape Town, South Africa; Clinical Research Centre, Faculty of Health Sciences, University of Cape Town, Anzio Road, Observatory 7925 Cape Town, South Africa; Division of Endocrinology and Diabetes, Chronic Disease Initiative in Africa (CDIA) Department of Medicine, Faculty of Health Sciences, University of Cape Town, Anzio Road, Observatory 7925 Cape Town, South Africa; Division of Hepatology, Department of Medicine, Faculty of Health Sciences, University of Cape Town, Anzio Road, Observatory 7925 Cape Town, South Africa; Department of Medicine, Faculty of Health Sciences, University of Cape Town, Anzio Road, Observatory 7925 Cape Town, South Africa

**Keywords:** *N*-acetylcysteine, Acetylcysteine, Drug-induced, Hepatitis, Liver, Liver failure, Non-paracetamol, Non-acetaminophen

## Abstract

**Background:**

Drug-induced liver injury (DILI) refers to acute or chronic liver injury that may occur as a consequence of using drugs and herbal or dietary supplements. Specific therapies for DILI are limited. There is considerable evidence for efficacy and safety of *N*-acetylcysteine (NAC) in management of paracetamol-induced liver injury. More recently, research has explored the use of NAC in non-paracetamol drug-induced liver injury. It is important to summarise the evidence of NAC for non-paracetamol DILI to determine if NAC may be considered a therapeutic option in this condition.

**Methods/design:**

We will conduct a systematic review of the benefit and harm of NAC in non-paracetamol drug-induced liver injury. Primary and secondary outcomes of interest are pre-specified. Primary outcomes include all-cause mortality, mortality due to DILI, time to normalisation of liver biochemistry (e.g. return of alanine transaminase to <100 U/l and/or international normalized ratio (INR) <1.5) and adverse events. Secondary outcomes include transplantation rate, time to transplantation, transplant-free survival and duration of hospitalisation. We will include randomized controlled trials (RCTs) and prospective cohort studies. RCTs will contribute to the evaluation of safety and efficacy of NAC, whereas, the cohort studies will contribute exclusively to the evaluation of safety. We will search several bibliographic databases (including PubMed, Scopus, CINAHL, CENTRAL), grey literature sources, conference proceedings and ongoing trials. Following data extraction and assessment of the risk of bias, we will conduct a meta-analysis if feasible, as well as subgroup analyses. We will assess and explore clinical and statistical heterogeneity.

**Discussion:**

The aim of this review is to provide evidence on the effectiveness and safety of NAC in non-paracetamol DILI. We anticipate that the results could aid health care practitioners, researchers and policymakers in the decision-making regarding the use of NAC in patients with non-paracetamol DILI.

**Systematic review registration:**

PROSPERO CRD42014008771

**Electronic supplementary material:**

The online version of this article (doi:10.1186/s13643-015-0075-6) contains supplementary material, which is available to authorized users.

## Background

Drug-induced liver injury (DILI) refers to acute or chronic liver injury that may occur as a consequence of using drugs and herbal or dietary supplements [1, 2]. According to recent estimates, the yearly incidence of DILI is estimated to be between 14–19 cases per 100,000 [3, 4]. While this may suggest that the condition is uncommon, there is still a considerable potential for harm. In the USA, it is the most common cause of acute liver failure, with 11 % of cases due to idiosyncratic DILI [5]. Moreover, the true incidence of DILI may be underestimated due to diagnostic difficulty as well as underreporting [2].

A number of risk factors are thought to be associated with the development of DILI. In general, older age is a risk factor, with DILI occurring more commonly in adults compared with children [6]. While there seems to be a biological basis for age as a risk factor, it may also reflect that adults are more frequently exposed to potential hepatotoxins compared with children. However, age as a risk factor does not always hold true, such that for certain drugs, the risk is greater in children e.g. DILI caused by valproic acid is more common in children. Females appear to be at a greater risk compared to their male counterparts [7]. Certain genetic variations place individuals at risk of DILI due to specific drugs e.g. isoniazid DILI and *N*-acetyltransferase 2 gene polymorphism as well as the HLA-*B5701 genotype and flucloxacillin [8]. While these genetic variations have been shown to increase the risk for the development of DILI, they do not predict severity of injury. Pre-existing liver disease is a further independent risk factor, with this being observed in patients coinfected with viral hepatitis and tuberculosis who develop DILI in response to antiviral and antituberculous drugs [9, 10]. Furthermore, alcohol abuse and malnutrition are also risk factors associated with the development of DILI [2].

The general management of DILI consists of the discontinuation of the offending drug in combination with supportive treatment [2]. Patients often require prolonged hospital stays which may be costly to both patient and health service. Therapeutic re-challenge with the offending drug is generally not recommended but may be attempted in certain instances after a thorough consideration of the risks and potential benefits. There are specific therapies available for DILI caused by certain drugs. However, these are limited to carnitine for valproic acid and *N*-acetylcysteine (NAC) for paracetamol overdose [11, 12]. This limited availability highlights the need for further research into therapies for DILI.

NAC was first used as a treatment for paracetamol overdose in 1979 [13]. Since then, it has been firmly established as an effective and safe treatment for this condition [12]. NAC has also been shown to be safe and effective outside of paracetamol overdose. NAC has been evaluated as a treatment option for non-paracetamol acute liver failure in adults and paediatric patients. In a randomised clinical trial comparing NAC with placebo in adults with non-paracetamol acute liver failure, NAC was associated with an improvement in transplant-free survival in a subgroup of patients with grade 1 and grade 2 encephalopathy [14]. In a prospective study conducted in adults with non-paracetamol acute liver failure at a centre without the facility for transplantation, the use of NAC was associated with a mortality benefit [15]. In a retrospective study in paediatric patients with non-paracetamol acute liver failure, NAC was associated with a shorter hospital stay and improved survival post-transplantation [16]. Furthermore, in a case series of patients with DILI secondary to Amanita phalloides mushroom poisoning, 10 out of 11 patients recovered fully after receiving NAC in combination with other therapies [17].

NAC has also been evaluated for non-liver related clinical indications. These indications include its use as a mucolytic agent in pulmonary diseases, in the prevention of radio-contrast associated nephrotoxicity and for the treatment of certain ophthalmic conditions [18–21].

In paracetamol overdose, a form of non-idiosyncratic DILI, the pathogenesis underlying hepatotoxicity is fairly well understood. Here, the metabolism of paracetamol produces an excess of the hepatotoxic metabolite *N*-acetyl-p-benzo-quinone imine (NAPQI). NAPQI is normally inactivated by hepatic glutathione; however, glutathione is depleted in paracetamol overdose. This results in an accumulation of NAPQI with consequent hepatic cell injury and death. NAC is thought to replenish hepatic glutathione stores, which forms the basis for its efficacy in this condition [22]. In contrast, the mechanism underlying hepatotoxicity in idiosyncratic DILI does not involve glutathione depletion. However, the precise pathogenesis in idiosyncratic DILI is not clearly defined [23]. The proposed pathogenic mechanisms in idiosyncratic DILI include direct cell injury, immune mediated damage and mitochondrial injury. These mechanisms, especially those that lead to mitochondrial damage, have significant implications. Mitochondria are involved in protecting hepatocytes against oxidative stress from oxygen-free radicals in the liver. The damage and loss of mitochondria leads to an accumulation of oxygen-free radicals and subsequent oxidative cell damage. NAC may be of benefit in this context through its antioxidant effect [24, 25]. Furthermore, additional benefits of NAC in this context involve the improvement of systemic haemodynamics and tissue oxygen delivery, as well as other favourable effects on the injured liver [26, 27].

The aim of this systematic review is to review the evidence of safety and effectiveness including improvement in time, if any, to normalisation of liver function tests and of NAC in non-paracetamol drug-induced liver injury. NAC has already been established as a safe and effective treatment for paracetamol-induced liver injury. Recently, the research focus has shifted to investigating the use of NAC in non-paracetamol drug-induced liver injury. It is important to review the evidence of NAC safety and efficacy in this setting to determine if NAC may be considered as a treatment option in non-paracetamol drug-induced liver injury. The evidence from this research may then be used to inform the decisions made by policymakers, health care practitioners, as well as researchers in this area.

## Methods/design

This review protocol is registered in the PROSPERO International Prospective Register of systematic reviews, registration number CRD42014008771.

### Criteria for considering studies for this review

#### Types of studies

We will include randomized controlled trials (RCTs) and prospective cohort studies. RCTs will contribute to the evaluation of safety and efficacy of NAC, whereas, the cohort studies will contribute exclusively to the evaluation of safety.

#### Language and timing

No language and time restrictions will apply.

#### Types of participants

Human participants of any age diagnosed with non-paracetamol drug-induced liver injury and diagnosed according to recognised diagnostic criteria [28–31].

#### Types of interventions

Intervention, *N*-acetylcysteine administered intravenously or orally.

Control, placebo or standard of care (as described in the study) or alternative therapy.

There will be no restriction on dose, timing and route of administration of NAC.

#### Types of outcome measures

Results must include quantitative data for outcomes measured.

##### Primary outcomes

All-cause mortality, mortality due to DILI, time to normalisation of liver biochemistry (e.g. return of alanine transaminase to <100 U/l and/or international normalized ratio (INR) <1.5), adverse events (graded using the Common Terminology Criteria for Adverse Events) [32].

##### Secondary outcomes

Transplantation rate, time to transplantation, transplant-free survival, duration of hospitalisation.

### Search methods for identification of studies

We will perform a comprehensive search of databases and conference proceedings to identify all relevant studies available by October 2014, regardless of language or publication status. We will search both peer-reviewed journal articles and grey literature (unpublished, internal or non-reviewed papers and reports).

### Electronic searches

We will search the following electronic databases: Cochrane Library, Medline via PubMed, SCOPUS, Web of Science (SciELO), and EBSCO (CINAHL, Africa-Wide, Academic Search Premier). We will use both text words and medical subject heading (MeSH) terms. The literature search strategy will be adapted to suit each database. Briefly, we will use a combination of the following terms: *N*-acetylcysteine, Acetylcysteine, Drug-induced, Hepatitis, Liver, Liver Failure, Non-paracetamol, Non-acetaminophen.

The detailed search strategy is provided in Additional file 1.

### Conference proceedings

We will conduct a manual search of relevant abstracts or proceedings of the following conferences (2000 to present): American Association for the Study of Liver Diseases (AASLD) Drug-Induced Liver Injury Conference, AASLD-FDA-NIH-PhRMA-Hepatotoxicity Special Interest Group Conferences, European Association for the Study of Liver (EASL), The International Liver Congress and Digestive Diseases Week (DDW). If conference abstracts are not adequately comprehensive, we will use the information from these abstracts to search for the full text articles. We will attempt to contact the authors of the conference abstracts if we are unable to track down the full text articles. If we are unable to obtain the full text articles and contact the authors, we will list the studies as potentially relevant.

### Manual searches

We will obtain reference lists of relevant studies identified, and the full text articles reviewed for inclusion in the review will be checked for additional information.

#### Searching other sources

Grey Literature will include Google Scholar, SCOPUS for conference proceedings. www.opengrey.eu and www.greylit.org. For ongoing studies, we will search the Pan African National Clinical Trials Registry (PACTR), World Health Organisation (WHO) International Clinical Trials Registry Platform (ICTRP), and ClinicalTrials.gov and NHS Clinical Trials. Individuals and organisations working in the field of drug-induced liver injury will be consulted for information regarding unpublished data and work in progress.

### Data collection and analysis

The methods for data collection and analysis will be based on the Cochrane Handbook of Systematic Reviews for Interventions [33].

### Selection of studies

Two review authors (MFC and NK) will independently review all relevant material identified from the above search. After reading the titles and abstracts of the identified articles, we will acquire the full text articles of all citations deemed to meet the inclusion criteria. These articles will be independently inspected to verify that they meet the pre-specified inclusion criteria. We will resolve disagreements between the two reviewers regarding study eligibility through discussion with a third author (KC). For all studies excluded by the assessors, we will describe the reasons for exclusion.

### Data extraction and management

MFC and NK will use a standardised data extraction form to extract data from the included studies and to assess the study quality. Extracted information will include administrative details, verification assessment of the diagnosis of DILI, details of the intervention, details of comparators, details of outcomes and information for assessment of the risk of bias. A pilot data extraction will be performed using the data extraction form, and the form will be modified if required. Any discrepancies will be resolved via discussion of the original articles with a third author (KC). We will request missing data from study authors. References will be managed using Mendeley Desktop reference manager and data will be analysed using Review Manager 5.3 (RevMan5) software. MFC and NK will both enter data and conduct crosschecks to ensure that there are no data entry errors.

### Assessment of risk of bias in included studies

MFC and NK will independently assess the risk of bias in each of the included studies. The assessment will include information on the following: sequence generation, allocation concealment, blinding, incomplete outcome data or missing data, selective outcome reporting, other sources of bias and overall risk of bias. Each methodological component will be assessed, and the RCTs will be described as having a low, unclear or high risk of bias, as per the Cochrane Handbook of Systematic Reviews of Interventions [33]. The Newcastle-Ottawa Scale (NOS) for assessing the quality of nonrandomized studies in meta-analyses tool will be used for assessing the risk of bias of the included cohort studies [34]. More specifically, we will use an adapted version of the modified NOS (see Additional file 2) to assess the risk of bias of the included cohort studies [35]. This modified NOS includes seven questions amongst four domains of risk assessment: methods for selecting study participants (selection bias), methods to control for confounding (performance bias), statistical methods (detection bias) and methods of exposure and outcome assessment (information bias). Risk of bias will be measured using a scale ranging from 0 (high risk of bias) to 3 (low risk of bias), and question-specific descriptions including examples of varying degrees of bias are included. Items from the original NOS pertaining to adequacy of follow-up, selection of participants (representativeness of cohort) and assessment of outcomes will be retained in the adapted modified NOS.

The two authors will resolve disagreements in the assessment of risk of bias by discussion and consensus, consulting KC to resolve any persistent disagreements.

### Measures of treatment effect

Data will be analysed using RevMan5. The type of outcomes may include dichotomous, continuous and time-to-event data. For dichotomous data, a summary statistic will be calculated (e.g. odds ratio and risk ratio) with accompanying confidence interval (e.g. 95 % CI). For continuous data, a summary statistic such as a mean difference or standardised mean difference will be calculated. Two methods of summarising the time-to-event data will be considered. The first will use the methods of survival analysis and express the intervention effect as a hazard ratio. For the second method, the time-to-event data may be analysed as dichotomous data if the status of all study participants at a fixed time point are known and further summarised as an odds ratio or risk ratio with accompanying confidence interval. Every effort will be made to contact the original authors or investigators of the selected articles to assist with missing or incomplete data.

### Dealing with missing data

In the cases of absent or incomplete evidence found in the included studies, authors will be contacted for further information. We will report unclear issues as presented rather than make assumptions. Should they be necessary, we will be explicit about assumptions made.

### Data synthesis, assessment/investigation of heterogeneity

Heterogeneity will be assessed by inspecting forest plots initially then through the Cochran’s chi-square test using a 10 % level of significance cut-off (due to the low power of the test) and the I-square statistic (*I*^2^) where values will be evaluated as follows:

- 0–40 % = might not be important
- 30–60 % = moderate
- 50–90 % = substantial
- 75–100 % = considerable

Where heterogeneity is statistically significant, subgroup analysis using the variables of age group, sex and setting (e.g. geographical region), as well as sensitivity analysis, will be conducted to explore the potential sources of heterogeneity. Symmetry of funnel plots will be used to assess for publication or selective reporting bias.

We will attempt primary meta-analyses of the included RCTs for both effectiveness and harm outcomes. If meta-analysis of RCTs is feasible, a random effects model will be constructed. We plan to quantify the statistical reliability of data in the cumulative meta-analysis by undertaking sequential analysis. Should small study effects be found, we will conduct a meta-regression on small study effects. If the identified RCTs are of substantial heterogeneity rendering meta-analysis not feasible, the findings will be presented in narrative form and will include relevant tables and figures to aid in data presentation. We will consider conducting separate secondary meta-analyses for prospective cohort studies limited to the outcomes of harm. If this is not feasible, the findings from the included cohort studies will be presented in a narrative form. All authors will contribute to the narrative review.

In addition to evaluating all DILI participants, we also plan to explore differences in outcomes between the following subgroups: sex, age strata, geographical region, diagnostic certainty of DILI and exclusion of other possible causes, aetiology of DILI (e.g. antituberculous, HIV antiretrovirals, antiepileptics, herbal supplements), coma grade, severity of DILI (using severity scales such as Drug-Induced Liver Injury Network 5-point scale where severity of liver injury is based upon the presence of jaundice, hospitalisation, signs of hepatic or other organ failure, ultimate outcome and graded as 1+ mild, 2+ moderate, 3+ moderate-severe, 4+ severe, 5+ fatal) and pattern of liver injury (hepatocellular, mixed and cholestatic). For the analyses of outcomes within subgroups, the same methods of analyses for measuring treatment effects as a whole will be applied.

We will use the grading of Recommendations Assessment, Development and Evaluation (GRADE) approach to assess the quality of evidence [36].

### Sensitivity analyses

A sensitivity analysis of the findings from primary meta-analysis is planned, and the aim is to determine whether the findings are robust to decisions made during the review process [32]. Amongst others, we will explore the impact of including or excluding particular studies and the chosen method for analysis. Lastly, we will also evaluate the impact of excluding studies deemed as having a high risk of bias.

### Presenting and reporting of results

This systematic review will be reported according to the Preferred Reporting Items for Systematic reviews and Meta-Analyses (PRISMA) statement [37].

## Discussion

This review will provide evidence on the effectiveness and safety of NAC in non-paracetamol DILI. We anticipate that the findings could aid health care practitioners and policymakers in the decision-making regarding the use of NAC in patients with non-paracetamol DILI. Furthermore, the findings may benefit researchers by providing guidance for the focus of future research through the identification of gaps in the existing evidence and advise on the conduct of future high-quality research through the identification of the shortcomings in previously conducted research.

## Additional files

Additional file 1:
**Electronic search strategy.** This describes the electronic search strategy used in searching the electronic databases. Additional file 2:
**Modified Newcastle-Ottawa Scale.** This describes an adapted version of a modified Newcastle-Ottawa Scale for the risk of bias assessment of included cohort studies.

## Abbreviations

ALT: alanine aminotransferase
DDW: Digestive Diseases Week
DILI: drug-induced liver injury
EASL: European Association for the Study of Liver
ICTRP: International Clinical Trials Registry Platform
INR: international normalized ratio
NAC: *N*-acetylcysteine
NAPQI: *N*-acetyl-p-benzoquinone imine
PACTR: Pan African National Clinical Trials Registry
RCT: randomized controlled trial
RevMan: Review Manager
WHO: World Health Organization
