# Supplementary material for: N-acetylcysteine for non-paracetamol drug-induced liver injury: a systematic review protocol
Source: Syst Rev. 2015 Jun 12;4:84. doi: 10.1186/s13643-015-0075-6 (PMC4470061; doi:10.1186/s13643-015-0075-6)
Supplement: Additional file 1: — Electronic search strategy. This describes the electronic search strategy used in searching the electronic databases. [file 13643_2015_75_MOESM1_ESM.pdf]

Additional file 1: Electronic search strategy

Search	Query
#5	Search (#3 AND #4)
#4	Search (acetylcysteine[mh] OR acetylcysteine[tiab] OR n-acetylcysteine[tiab] OR n-acetyl-L-cysteine[tiab] OR n-acetylcystine[tiab] OR acetyl cysteine[tiab] OR n-acetyl cysteine[tiab])
#3	Search (#1 OR #2)
#2	Search (liver failure[mh] OR liver failure[tiab] OR hepatic failure[tiab] OR hepatic injur*[tiab])
#1	Search (chemically-induced liver failure OR drug-induced liver injury[mh] OR drug induced liver injur*[tiab] OR drug induced liver disease*[tiab] OR drug induced hepatitides[tiab] OR drug induced hepatitis[tiab] OR toxic hepatitides[tiab] OR toxic hepatitis[tiab])
