# Supplementary material for: N-acetylcysteine for non-paracetamol drug-induced liver injury: a systematic review protocol
Source: Syst Rev. 2015 Jun 12;4:84. doi: 10.1186/s13643-015-0075-6 (PMC4470061; doi:10.1186/s13643-015-0075-6)
Supplement: Additional file 2: — Modified Newcastle-Ottawa Scale. This describes an adapted version of a modified Newcastle-Ottawa Scale for the risk of bias assessment of included cohort studies. [file 13643_2015_75_MOESM2_ESM.pdf]

Additional file 2: Adapted version of a modified Newcastle-Ottawa Scale for single use in specific context

Modified Newcastle-Ottawa Scale (NOS)

Legend

0 = Definitely no (high risk of bias)
1 = Mostly no
2 = Mostly yes
3 = Definitely yes (low risk of bias)

Domain of evaluation: Methods for selecting study participants (*i.e. Selection bias)*

Is the source population (cases, controls, cohorts) appropriate and representative of the population of interest?

0	1	2	3
(high risk of bias)			(low risk of bias)

Example of low risk of bias: A consecutive sample or random selection from a population that is representative of the condition under study.

Example of moderate risk of bias: A consecutive sample or random selection from a population that is not highly representative of the condition under study.

Example of high risk of bias: The source population cannot be defined or enumerated (i.e. volunteering or self-recruitment).

Domain of evaluation: Methods to control confounding (*i.e. Performance bias)*

Is the sample size adequate and is there sufficient power to detect a meaningful difference in the outcome of interest?

0	1	2	3
(high risk of bias)			(low risk of bias)

Example of low risk of bias: Sample size was adequate and there was sufficient power to detect a difference in the outcome.

Example of high risk of bias: Sample size was small and there was not enough power to test outcome of interest.

Did the study identify and adjust for any variables or confounders that may influence the outcome?

0 1 2 3

(high risk of bias) (low risk of bias)

<u>Example of low risk of bias:</u> The study identified and adjusted for all possible confounders that may influence estimates of association between exposure and outcome (*i.e. Was the patient being treated for a medical condition such as chronic pain and was being prescribed opioids while on methadone treatment?*)

<u>Example of moderate risk of bias:</u> The study identified and reported possible variables that may influence the outcome but did not explore the interaction.

<u>Example of high risk of bias:</u> The study either did not report any variables of influence or acknowledge variables of influence when it was clear they were present.

**Domain of evaluation:** Statistical methods (*i.e. Detection bias)*

Did the study use appropriate statistical analysis methods relative to the outcome of interest?

0	1	2	3
(high risk of bias)			(low risk of bias)

<u>Example of low risk of bias:</u> The study reported use of appropriate statistical analysis as required (*i.e. adjusting for an unbalanced distribution of a specific covariate among sexes, or correcting for multiple testing error*)

<u>Example of moderate risk of bias:</u> The study either used correct statistical methods but did not report them well, or used the incorrect methods but reported them in detail.

<u>Example of high risk of bias:</u> The study did not use appropriate statistical analysis as required (*i.e. did not adjust for an unbalanced distribution of a specific covariate among sexes, or correct for multiple testing error when necessary*) or did not report them adequately.

Is there little missing data and did the study handle it accordingly?

0	1	2	3
(high risk of bias)			(low risk of bias)

<u>Example of low risk of bias:</u> The study acknowledged missing data to be less than 10% and specified the method of handling it.

<u>Example of moderate risk of bias:</u> The study either had greater than 15% but they specified the method they used to handle it.

<u>Example of high risk of bias:</u> The study had greater than 15% missing data and did not handle it at all.

**Domain of evaluation:** Methods for measuring outcome variables (*i.e. Information bias)*

Is the methodology of the outcome measurement explicitly stated and is it appropriate?

0<br>(high risk of bias)	1	2	3<br>(low risk of bias)

Example of low risk of bias: The study provides a detailed description of the outcome measure(s) which are appropriate for the outcome of interest.

Example of moderate risk of bias: The study provides a somewhat complete description of outcome measurements and they are justified.

Example of high risk of bias: The study provides limited information on the methods of measuring the outcome and the measure is not appropriate considering the outcome.

Is there an objective assessment of the outcome of interest?

0<br>(high risk of bias)	1	2	3<br>(low risk of bias)

Example of low risk of bias: The study used objective methods to discern the outcome status of participants (*i.e. laboratory measurements, medical records*).

Example of moderate risk of bias: The study relied on subjective data as the primary method to discern outcome status of participants (*i.e. self-report*).

Example of high risk of bias: The study had limited reporting about assessment of outcomes.
